# The Influence of Balneotherapy Using Salty Sulfide–Hydrogen Sulfide Water on Selected Markers of the Cardiovascular System: A Prospective Study

**DOI:** 10.3390/jcm13123526

**Published:** 2024-06-16

**Authors:** Tomasz Zapolski, Wojciech Kornecki, Andrzej Jaroszyński

**Affiliations:** 1 Department of Cardiology, Medical University of Lublin, 20-093 Lublin, Poland; 2 Sanatorium “Włókniarz”, 28-100 Busko Zdrój, Poland; wojtek_kornecki@onet.pl; 3 Department of Internal Medicine and Family Medicine, Collegium Medicum, Jan Kochanowski University in Kielce, 25-369 Kielce, Poland; jaroszynskiaj@interia.pl

**Keywords:** balneotherapy, sulfide–hydrogen sulfide brine, osteoarticular disorders, cardiovascular disease, troponin, NT-proBNP

## Abstract

**Background:** The sulfide–hydrogen sulfide brine balneotherapy (HSBB), including a combination of dissolved hydrogen sulfide (H_2_S) gas, inorganic sulfur ions (S^2−^), and hydrosulfide ions (HS^−^), is one of the most important and most effective forms of spa treatment in patients with osteoarticular disorders (OADs). Some cardiovascular diseases (CVDs) are often considered to be contraindications to HSBB since the presence of thiol groups may lead to an increased quantity of reactive oxygen species (ROS), which damage the vascular endothelium, and endothelial dysfunction is considered to be the main cause of atherosclerosis. However, there are a number of literature reports suggesting this theory to be false. H_2_S is a member of the endogenous gaseous transmitter family and, since it is a relatively recent addition, it has the least well-known biological properties. H_2_S–NO interactions play an important role in oxidative stress in CVDs. The general objective of this study was to assess the cardiovascular safety of HSBB and analyze the effect of HSBB on selected cardiovascular risk markers. **Methods:** A total of 100 patients at the age of 76.3 (±7.5) years from the Włókniarz Sanatorium in Busko-Zdrój were initially included in the study. The following parameters were assessed: age, sex, height, body weight, body surface area (BSA), body mass index (BMI), systolic (SBP) and diastolic blood pressure (DBP), heart rate, the diagnosis of OAD that was the indication for balneotherapy, creatinine (CREAT), glomerular filtration rate (GFR), lipid panel, C-reactive protein (CRP), uric acid (UA), and fibrinogen (FIBR) and cardiovascular markers: (cardiac troponin T (cTnT), N-terminal pro-B-type natriuretic peptide (NT-proBNP). **Results:** A significant decrease in DBP and a trend towards SBP reduction were observed over the course of the study. A significant decrease was observed in CRP levels decreasing from 2.7 (±3.6) mg/L to 2.06 (±1.91) mg/L, whereas FIBR rose significantly from 2.95 (±0.59) g/L to 3.23 (±1.23) g/L. LDL-C levels decreased slightly, statistically significant, from 129.36 (±40.67) mg/dL to 123.74 (±36.14) mg/dL. HSBB did not affect the levels of evaluated cardiovascular biomarkers, namely NT-proBNP (137.41 (±176.52) pg/mL vs. 142.89 (±182.82) pg/mL; *p* = 0.477) and cTnT (9.64 (±4.13) vs. 9.65 (±3.91) ng/L; *p* = 0.948). A multiple regression analysis of pre-balneotherapy and post-balneotherapy values showed cTnT levels to be independently correlated only with CREAT levels and GFR values. None of the assessed parameters independently correlated with the NT-proBNP level. **Conclusions:** HSBB resulted in a statistically significant improvement in a subclinical pro-inflammatory state. HSBB has a beneficial effect in modifying key cardiovascular risk factors by reducing LDL-C levels and DBP values. HSBB has a neutral effect on cardiovascular ischemia/injury. Despite slightly elevated baseline levels of the biochemical marker of HF (NT-proBNP), HSBB causes no further increase in this marker. The use of HSBB in patients with OAD has either a neutral effect or a potentially beneficial effect on the cardiovascular system, which may constitute grounds for further studies to verify the current cardiovascular contraindications for this form of therapy.

## 1. Introduction

Sulfide–hydrogen sulfide brine balneotherapy (HSBB) is one of the most important and most effective forms of spa treatment in patients with osteoarticular disorders (OADs). Sulfur-rich healing waters contain active forms of sulfur (S), including a combination of dissolved hydrogen sulfide (H_2_S) gas, inorganic sulfur ions (S^2−^), and hydrosulfide ions (HS^−^) in a suitable ratio. The occurrence of these three sulfur compounds in water is pH-dependent. This is due to the fact that the sulfate ion (SO4^2−^) only occurs in highly alkaline water, and H_2_S, which takes the gaseous form, only occurs in highly acidic water; therefore, in spring waters with a pH ranging between 6 and 8, there is a mixture of H_2_S and HS^−^ [1]. HSBB in patients with degenerative joint disease has been demonstrated to have an undoubtedly beneficial effect on alleviating pain in OAD patients and improving their fitness levels. [2]. The key aspect of this method lies in reducing the underlying inflammatory process. Studies have shown reduced levels of classic inflammatory markers to be associated with an improved musculoskeletal condition in OAD patients. However, even more importantly, the effectiveness of HSBB in reducing inflammation has been also demonstrated in recent studies with the use of sensitive biochemical indices designated for accurately assessing the severity of inflammation [3].

Along with nitric oxide (NO) and carbon monoxide (CO), H_2_S is a member of the endogenous gaseous transmitter family and, since it is a relatively recent addition, it has the least well-known biological properties [4]. Having been considered toxic and potentially lethal for centuries, these gases are currently believed to be important endogenous, cytoprotective modulators of many physiological functions. Studies in vascular tissues have shown that H_2_S acts at the level of nitric oxide synthase (NOS), and NO affects the activity of cystathionine γ-lyase (CSE). H_2_S (or molecules derived from it) interact with NO of vascular origin and vice versa [5]. In this way, the concept of mutual molecular-physiological coupling between H_2_S and NO was developed. The role of the influence of H_2_S-NO interactions on the cardiovascular system has been analyzed in many physiological and pathophysiological approaches [5,6,7,8,9,10,11]. A positive inotropic/lusitropic effect of the H_2_S-NO interaction product, HNO, has been demonstrated, which makes it an attractive addition to the current therapeutic arsenal for the treatment of patients with acute decompensated congestive heart failure (HF). [12]. Numerous potential cardioprotective effects of the H_2_S-NO duo have been found, protecting the heart against ischemic damage. It has been confirmed that reducing endogenous H_2_S production increases the size of myocardial infarction (MI), suggesting an important role of endogenous H_2_S in maintaining proper heart function. Inhibiting NO production with NG-nitro-L-arginine methyl ester (L-NAME), a non-selective NO synthase inhibitor, significantly reduces the cardioprotective effect of ischemic preconditioning with H_2_S [13].

Both OAD and cardiovascular disease (CVD) have an inflammatory component. Inflammation is known to be inextricably associated with a hypercoagulable (or prothrombotic) state. Some cardiovascular diseases (CVDs) are often considered to be contraindications to HSBB, since the presence of thiol groups may lead to an increased quantity of reactive oxygen species (ROS), which damage the vascular endothelium, and endothelial dysfunction is considered to be the main cause of atherosclerosis [14]. From the clinical point of view, the concerns associated with using this form of balneotherapy stem from the observation that S^2−^ and H_2_S induce vasodilation (which affects various blood vessels, including the aorta and the portal vein) and cause a transient lowering of blood pressure [15]. However, there are a number of literature reports suggesting this theory to be false. Endogenously produced H_2_S is responsible for inducing a variety of physiologically beneficial effects in various systems of the mammalian body. H_2_S–NO interactions play an important role in oxidative stress in CVD. H_2_S is a well-known antioxidant and has been shown to protect endothelial function under acute oxidative stress via direct oxygen (O_2_) uptake [10]. These data clearly indicate potential anti-ischemic and cardioprotective effects of H_2_S–NO balance for the heart muscle, which is consistent with the results of animal studies [16]. Moreover, H_2_S–NO interactions play an important role in myocardial contractility regulation, since H_2_S was shown to reverse the negative inotropic and lusitropic effects of high NO levels [11]. Consequently, these data indicate that H_2_S may help in HF prevention. 

Apart from the pathophysiological evidence (mentioned above) that casts doubt on the supposed harmfulness of this form of balneotherapy to the cardiovascular system, there is some clinical evidence to further dispel any such reservations. HSBB and balneotherapy with iodine–bromine brines improve lower limb blood perfusion and have a beneficial effect on some properties of blood in patients with peripheral artery disease (PAD) [17]. This improvement may be associated with improved endothelial function, which allows for a better use of the relaxation properties of blood vessels. Research into H_2_S suggests its potential to protect against cardiovascular disease. The cysteine-derived gasotransmitter H_2_S has interesting vasorelaxant, cytoprotective, antioxidant, and anti-inflammatory properties [18]. The success of H_2_S-based solutions remains uncertain and there are currently no clinically approved molecules exploiting its therapeutic potential [18]. In addition to the beneficial hemodynamic effect, HSBB may also improve some parameters of fat metabolism, which was demonstrated in studies in patients with osteoarthritis (OA) [17]. To date, there have not been any studies to assess the effect of biologically active forms of sulfur used during balneotherapy in clinical conditions on objective markers of myocardial injury and HF.

### Aim of the Study

The aim of the study was to assess the cardiovascular safety of HSBB in terms of ischemic cardiac injury and the development of HF, taking into account cardiovascular risk factors, including prothrombotic and pro-inflammatory states.

## 2. Material and Methods

### 2.1. Characteristics of Healing Water

This study was conducted in patients from the Włókniarz Sanatorium in Busko-Zdrój, Poland. The Włókniarz Sanatorium has its own source of unique healing water. The “Dobrowoda G-1” source derives from the Dobrowoda water reservoir resting 165–228 m underground. This water was recognized as healing water by the Regulation of the Council of Ministers of 8 April 2008 (Journal of Laws 08.66.404), and it also meets the criteria specified in paragraph 6, Section 2 of the “Geological and mining law” Act. The water contains a total of 14,483.73 mg/L of dissolved minerals, with sodium chloride and sulfate dominating. The specific components of water from the Dobrowoda G-1 intake are S compounds in the form of S^2+^, HS^−^ + H_2_S at a concentration of 58.7 mg/L, iodides at a concentration of 2.8 mg/L, and bromides at a concentration of 8.6 mg/L. The main cations are Na^+^—4090.00 mg/L, K^+^—106.00 mg/L, Mg^2+^—352.40 mg/L, and Ca^2+^—531.00 mg/L. The main anions are Cl^−^—6204.00 mg/L, SO_4_^2−^—2716.00 mg/L, and HCO^3−^—366.10 mg/L. The physical properties of water include the temperature—16.5 °C, pH—7.10, electrical conductivity—22.3 mS/cm, odor—hydrogen-sulfide, and taste—salty and bitter. The water directly extracted from the intake is clear and colorless, and gas pearls (H_2_S) are formed in it. When heated, sulfide water used for bathing may have a color ranging from yellow-greenish to gray due to the oxidation of iron contained in the sulfide water, or slightly cloudy due to the oxidation of H_2_S to colloidal S.

### 2.2. Study Group and Balneological Procedures

A total of 100 patients from the Włókniarz Sanatorium in Busko-Zdrój were initially included in the study. These patients underwent HSBB, with 97 patients eventually completing the study and having their data analyzed. Indications for sanatorium treatment were OADs: spine osteoarthritis (SOA), osteoarthritis (OA), and rheumatoid arthritis (RA). The patients underwent 2 weeks of treatment in sulfide–hydrogen sulfide brine at 32–36 °C for 10–15 min per treatment throughout their rehabilitation regimen. The following procedures were performed on the patients. 

1. The so-called S baths—fully immersing the body in a bathtub in sulfur water up to the middle of the chest (semi-lying position) and not immersing deeper. Treatment duration—15 min. The treatment is performed once a day, 6 times a week. 

The sulfide half-baths—immersing the body in a bathtub in sulfur water “waist-deep” (sitting position) and not immersing deeper. Treatment duration—15 min. The treatment is performed once a day, 6 times a week.

The partial sulfide baths (sulfide baths)—sitting position on a chair, immersing the upper limbs up to the shoulders and lower limbs up to the knees, or only the upper or only the lower limbs in special containers with sulfide water. It is used when there are health contraindications to bathing in the bathtub, e.g., various types of skin lesions and skin damage, large varicose veins of the lower limbs or anus, and when the patient does not want to enter the bathtub.

### 2.3. Data Collection

- Demographic data: age [years] and sex [male/female];
- Anthropometric data: height [cm], body weight [kg], body surface area (BSA) [m^2^], body mass index (BMI) [kg/m^2^];
- Systolic blood pressure (SBP) [mm Hg] and diastolic blood pressure (DBP) [mm Hg];
- Heart rate (HR) [beats/min] and any possible arrhythmias during the course of the study;
- Data from medical records, specifically the diagnosis of OAD that was the indication for balneotherapy and cardiovascular comorbidities;
- Current OAD and CVD medications;
- Laboratory parameters.

Prior to and after the allotted course of HSBB, each patient had a blood sample collected for laboratory tests. In patients, blood for biochemical tests was collected from peripheral veins before the start of treatments using salty sulfide–hydrogen sulfide water and after the end of the stay. The first ml of blood was discarded, and subsequent portions were collected into test tubes. To obtain serum for determinations after coagulation, the samples were centrifuged for 15 min, 3000–5000. The test tube containing blood intended for N-terminal pro-B-type natriuretic peptide (NT-proBNP) determination was centrifuged for an hour according to the manufacturer’s recommendations. Cardiac troponin T (cTnT), measured via a highly sensitive method, was determined from whole blood collected with an anticoagulant (ethylenediaminetetraacetic acid). Biochemical tests apart from NT-proBNP and cTnT were performed using standard analyzers: ADVIA Centaur (Bayer—Bayer Health-Care Diagnostics, Tarrytown, New York, NY, USA). 

The following parameters were evaluated to satisfy the individual study objectives:

- Cardiovascular markers.
- Biochemical markers of the cardiovascular system regarding ischemia: cTnT concentration [ng/L] in whole blood was determined through electrochemiluminescence using the Elecsys 2010 analyzer from Roche (Roche Diagnostics GmbH, Mannheim, Germany). The cTnT cutoff point for this method according to the fourth universal definition of myocardial infarction is 14 ng/L (according to the definition, myocardial damage is defined as the detection of increased cTnT concentration in the blood above the upper limit of the reference range set at the 99th percentile in a healthy population) [19,20].
- Cardiovascular biochemical markers for heart failure: NT-proBNP. NT-proBNP [pg/mL] in blood plasma was determined through an enzyme-linked immunosorbent assay (ELISA) using a kit from Biomedica (Biomedica, Bratislava, Slovakia). The cutoff point for HF diagnosis is 125 pg/mL [21].
- Inflammatory and prothrombotic markers: C-reactive protein (CRP) [mg/L], uric acid (UA) [mg/dL], and fibrinogen (FIBR) [g/L].
- Renal function parameters: creatinine (CREAT) [mg/dL] and glomerular filtration rate (GFR) [mL/min/m^2^]. GFR was calculated using the MRDR method, according to the formula eGFR = 175 × [S_CREAT_]^−1.154^ × [age]^−0.203 ×^ 0.742 [if female] × 1.212 [if black] [22].
- Cardiovascular risk markers based on a lipid panel, including total cholesterol (TCHOL) [mg/dL], low-density lipoprotein cholesterol (LDL-chol) [mg/dL], high-density lipoprotein cholesterol (HDL-chol) [mg/d], and triglycerides (TGC) [mg/dL]. LDL-chol concentration was calculated based on the Friedewald–Levy–Fredrickson formula: LDL-chol = TCHOL − (HDL-chol + TGC/5) [23].

### 2.4. Statistical Analysis

The resulting data underwent a statistical analysis with the use of Statistica 12.0 software (StatSoft Inc.: Tulsa, OK, USA). Descriptive statistics were used to describe quantitative and qualitative data and the clinical variables of the study groups. Continuous variables were expressed as ranges, means, and standard deviations. The distribution of variables was assessed and tested for normality with the Shapiro–Wilk test. Student’s *t*-test for paired variables (pre- and post-balneotherapy values) was used to assess inter-group differences in paired quantitative variables of normal distribution. Linear correlation (with the use of the Pearson correlation coefficient) was used to calculate correlations between any two continuous variables, and the results were presented as numerical values. The correlations found between those markers and indices that were the most relevant for the study objectives were additionally presented in a graphic form. Linear regression (multiple stepwise linear regression) was used in order to identify potential independent predictive factors. The significance level cutoff was set at a *p*-value of 0.05.

## 3. Results

### 3.1. Demographic and Clinical Characteristics of the Study Group

**Figure 1:**
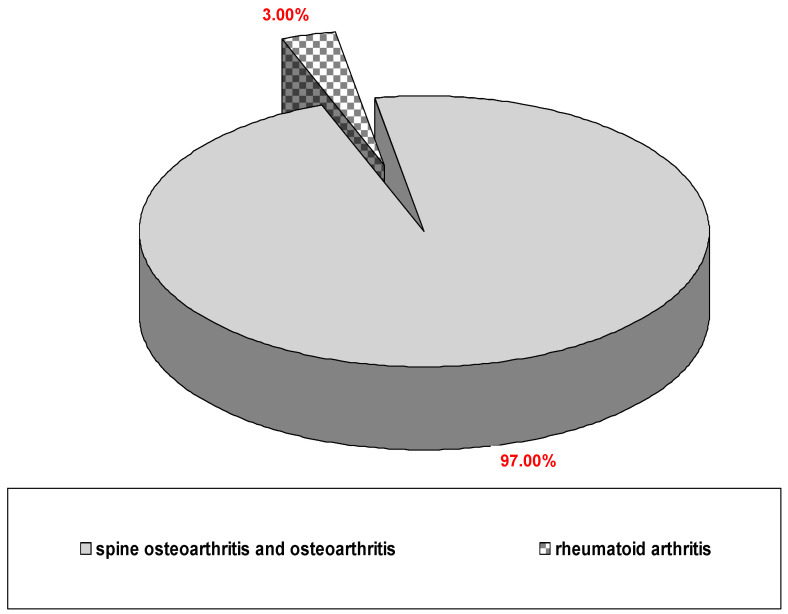
Diagnoses that constitute an indication for rehabilitation.

**Figure 2:**
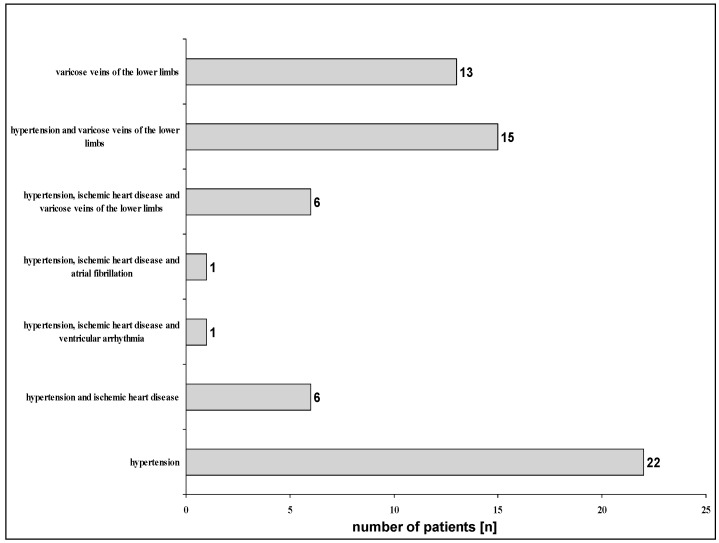
CVDs in the study group.

The study included a group of 100 patients aged 43 to 85, with an average age of 76.3 (±7.5). It included 64 women and 36 men. The basic demographic parameters of the study group are given in Table 1. The study group was very uniform in terms of indications for sanatorium rehabilitation and was dominated by patients with SOA and OA. (Figure 1). Only three patients had concurrent RA as a diagnosis, which indicated a medical recommendation for rehabilitation (Figure 1). When it comes to CVDs, patients with hypertension and ischemic heart disease (IHD) predominated; quite often, these two diseases occurred simultaneously (Figure 2). Varicose veins of the lower limbs were often found accompanying previous entities or occurring separately (Figure 2). Only one person reported paroxysmal atrial fibrillation (AF) in the interview. Similarly, ventricular arrhythmias occurred in only one patient. Arrhythmias have always occurred as abnormalities coexisting with hypertension and IHD (Figure 2). Therefore, in terms of CVDs, the group should also be considered homogeneous. This applies especially to cardiac arrhythmias, as the people included in the study, apart from the two cases mentioned, had no history of arrhythmias. 

### 3.2. Laboratory Data before and after HSBB

A significant decrease in DBP and a trend towards SBP reduction were observed over the course of the study (Table 2). However, the HR showed no significant changes over the course of the study (Table 2). During HSBB, no cases of any arrhythmia, including AF, were observed in the study group. There was no exacerbation of IHD, including no cases of acute coronary syndrome (ACS). Moreover, no cases of venous thromboembolism (VT), including venous thrombosis and pulmonary embolism, were observed during balneotherapy treatment.

The comparison of biochemical parameters before and after HSSB revealed numerous interesting observations. A significant decrease was observed in inflammatory marker levels, with the classic marker CRP decreasing from 2.7 (±3.6) mg/L to 2.06 (±1.91) mg/L (Table 2). However, the levels of another acute-phase protein, FIBR, rose significantly from 2.95 (±0.59) g/L to 3.23 (±1.23) g/L (Table 2). There were also interesting changes in lipid profiles. TG levels increased significantly from 121.37 (±56.30) mg/dL to 142.92 (±69.43) mg/dL, whereas LDL-C levels decreased slightly, though in a statistically significant way, from 129.36 (±40.67) mg/dL to 123.74 (±36.14) mg/dL (Table 2). HDL-C also decreased from its baseline level of 59.58 (±15.66) mg/dL to its post-HSBB level of 57.21 (±14.28) mg/dL (Table 2). In contrast, TCHOL levels did not change in a statistically significant way over the course of the study (Table 2).

**Figure 3:**
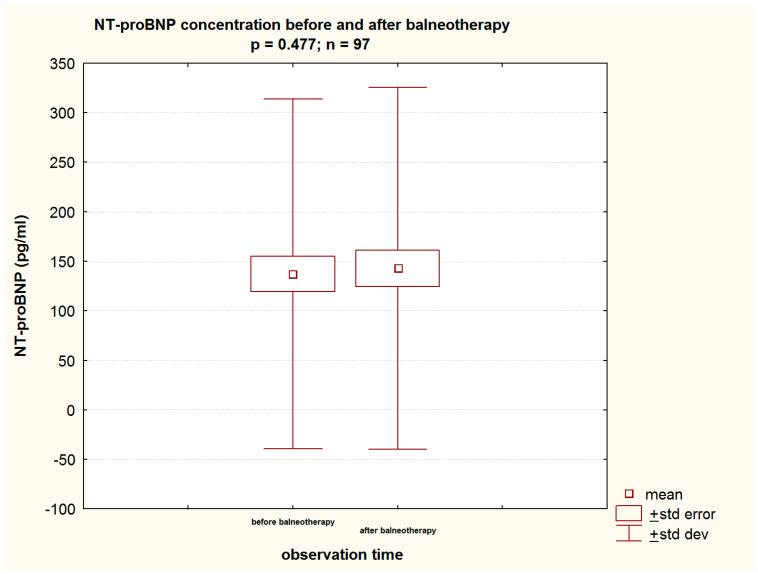
The level of NT-proBNP before and after balneotherapy in the study group.

**Figure 4:**
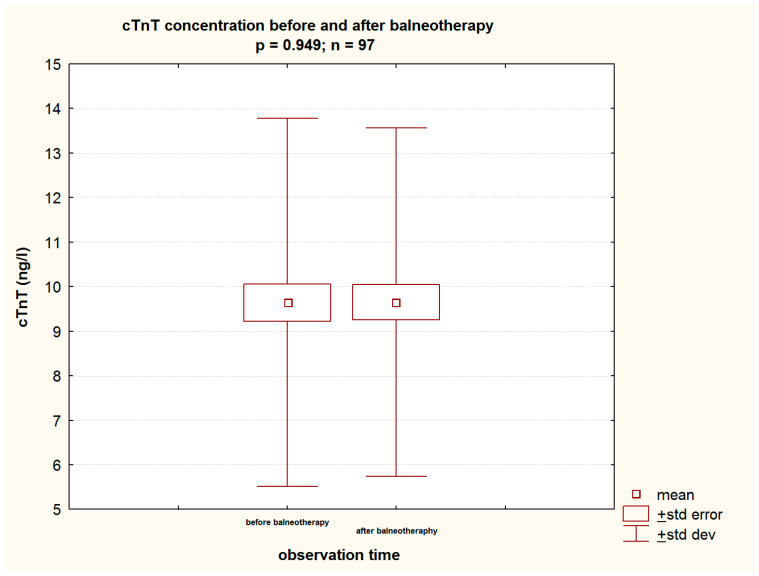
The level of cTnT before and after balneotherapy in the study group.

In order to address the study objectives, it must be stated that HSBB did not affect the levels of evaluated cardiovascular biomarkers, namely NT-proBNP (137.41 (±176.52) pg/mL vs. 142.89 (±182.82) pg/mL; *p* = 0.477) (Table 2, Figure 3) and cTnT (9.64 (±4.13) vs. 9.65 (±3.91) ng/L; *p* = 0.948) (Table 2, Figure 4). Moreover, the evaluated renal function parameters (CREAT and GFR) remained unchanged throughout the course of the study (Table 2). 

### 3.3. Relationship between cTnT Concentration and Other Laboratory Parameters

**Figure 5:**
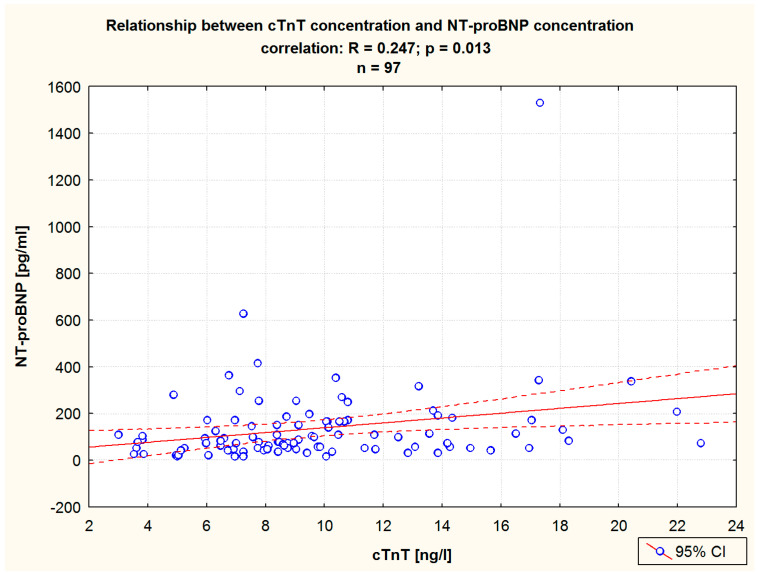
The relationship between the cTnT level and the NT-proBNP level before balneotherapy in the study group. Pearson correlation.

**Figure 6:**
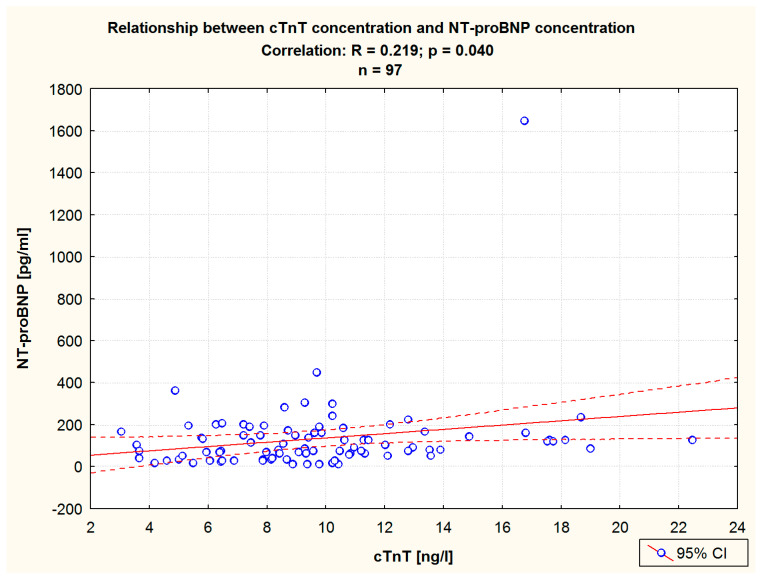
The relationship between the cTnT level and the NT-proBNP level after balneotherapy in the entire study group. Pearson correlation.

A Pearson correlation analysis of pre-balneotherapy cTnT levels showed this parameter to be correlated with the following ones: LDL-C, CREAT, GFR, (Table 3), and NT-proBNP (Table 3, Figure 5). An analogous analysis of post-HSBB values revealed cTnT levels to be significantly correlated with TC, LDL-C, CREAT, GFR, (Table 3), and NT-proBNP levels (Table 3, Figure 6). 

Next, to analyze the parameters independently related to the cTnT concentration before balneotherapy, a multiple regression model was constructed, which included the examined biochemical parameters that were significantly correlated with the cTnT value in the Pearson correlation analysis. The multiple regression analysis of pre-balneotherapy values showed cTnT levels to be independently correlated only with creatinine levels and GFR values (Table 4). An analogous multiple regression analysis of post-balneotherapy values showed that cTnT levels also correlated independently only with CREAT levels and GFR values (Table 5). 

### 3.4. The Relationship between the NT-proBNP Concentration and Other Laboratory Parameters

**Figure 7:**
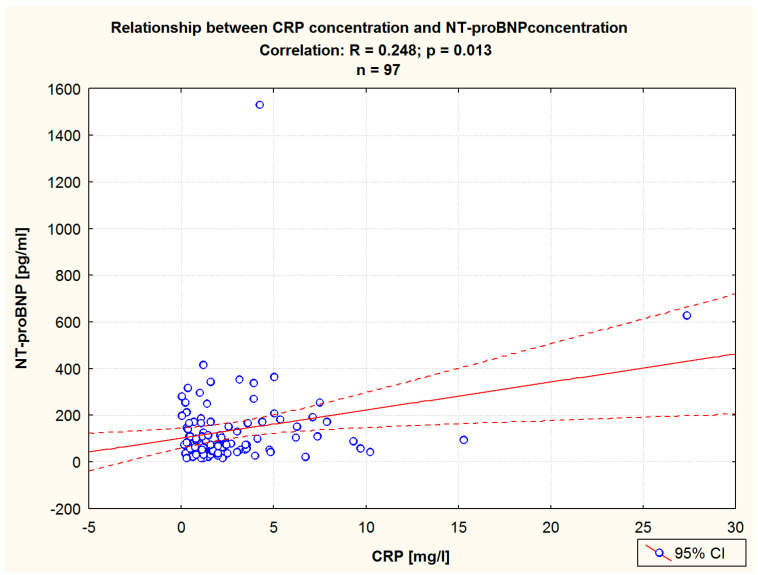
The relationship between the NT-proBNP level and the CRP level before balneotherapy in the study group. Pearson correlation.

The Pearson correlation analysis between the NT-proBNP level before HSSB revealed that it statistically correlates with CRP (Table 6, Figure 7) and cTnT (Table 6). An analogous correlation analysis using the Pearson method performed after balneological treatment showed that TGC and cTnT were significantly correlated with NT-proBNP concentration (Table 6). 

Next, to analyze parameters independently related to the NT-proBNP level before balneotherapy, a multiple regression model was constructed, which included all the tested biochemical parameters that were significantly correlated with the NT-proBNP value in the Pearson correlation analysis. Based on the multiple regression analysis, it was found that there are no independently associated parameters with the NT-proBNP concentration both before balneotherapy (Table 7) and after balneotherapy (Table 8).

## 4. Discussion

### 4.1. Blood Pressure and Heart Rate

Current research has shown that, regardless of other effects of HSBB, its beneficial effect was the reduction in blood pressure. As for SBP, it had no signs of statistical significance, although the *p*-value = 0.068 indicates a clear trend in this direction. However, DBP’s decrease was statistically significant (*p* < 0.00001). Both systolic and diastolic hypertension independently affect the risk of adverse cardiovascular events, which may have particularly important prognostic implications for patients undergoing HSBB [24]. There are limited data showing how treatment with salty sulfide–hydrogen sulfide waters affects blood pressure values. In this respect, the current research should be considered innovative. The importance of S^2−^ ions seems to be crucial here. The dilation of capillaries under the influence of histamine-like substances released in the skin by active forms of S contained in mineral waters causes the transfer of significant amounts of blood into the skin, which also lowers blood pressure in people with normal and high blood pressure [25]. Another piece of evidence is that the H_2_S-induced relaxation of vascular walls can be partially reduced through the removal of the vascular endothelium and/or under the influence of a NOS inhibitor such as L-NAME [7]. 

Arterial hypertension is mentioned as a potential contraindication to therapy with salty sulfur–hydrogen sulfide waters. The results of the current study seem to question this view, because after this form of balneotherapy, a statistically significant reduction in DBP and a trend towards a reduction in SBP were observed. Together with the data from the literature indicating the beneficial effect of balneotherapy on reducing blood pressure, this suggests the need to verify this contraindication. It seems advisable to specify that the only contraindication to HSSB should be uncontrolled or poorly controlled hypertension. When assessing the effects of balneotherapy, we should primarily remember the extremely favorable prognostic consequences resulting from the effective control of hypertension. These facts, together with data from the literature, encourage us to verify and mitigate the importance of hypertension as a contraindication to HSSB.

### 4.2. Lipids

Another equally important risk factor for CVD is lipid metabolism disorders. In the study group of patients undergoing HSSB, interesting changes in the lipid profile were noted. The TGC level increased significantly during the observation period from 121.37 mg/dL to 142.92 mg/dL, while the LDL-chol level decreased statistically significantly from 129.36 mg/dL to 123.7429 mg/dL. The level of HDL-chol changed similarly and decreased from 59.58 mg/dL before HSSB to 57.21 mg/dL after the end of the observation. However, the TCHOL level did not change statistically significantly during the observation period.

Several similar observations regarding lipid profile changes have already been observed in balneotherapy populations in other studies. Cencora et al. found that after treatments using sulfur baths, the levels of TCHOL and LDL-chol decreased [17]. It cannot be ruled out that the beneficial effect of sulfide–hydrogen sulfide saltwater therapy on LDL-chol observed in the current studies depends, at least in part, on the salt content. Studies assessing the effect of mineral waters on the extraction of bile acids and the levels of lipids and apolipoproteins in serum suggest that treatment with salt-rich spring waters reduces the level of LDL-chol in serum in people with mild hypercholesterolemia by increasing the fecal excretion of bile acids in sterol forms [26]. The results of Aksoy et al. published in 2022 indicate that the use of balneotherapy, including the use of S ions, positively modifies the lipid profile of rehabilitated patients [27]. This was expressed in a slight decrease in the levels of TCHOL, LDL-chol, and VLDL-chol and a slight increase in TGC concentration in patients at the end of spa therapy.

### 4.3. Subclinical Pro-inflammatory Condition

Both atherosclerosis, which is the cause of CVD, and OAD have an inflammatory cause in common. Therefore, a key element of current research in the context of the goals of this study is the behavior and inter-relationship of inflammation markers. Therefore, it is important to analyze changes in the concentrations of the biochemical markers of inflammation, which are the so-called acute-phase proteins: CRP and FIBR. Both of these biomarkers, apart from their pro-inflammatory aspect, play a significant role in stimulating and maintaining the prothrombotic state. Therefore, there is a potential pathophysiological pathway between the pro-inflammatory state associated with OAD and the prothrombotic state and further possible adverse cardiovascular consequences. These two elements were analyzed in detail in the context of the obtained research results. In the current study, an expected reduction in CRP values was noted from 2.7 (±3.6) mg/dL to 2.06 (±1.91) mg/dL, which should be interpreted as the expected anti-inflammatory effect of sulfur–hydrogen sulfide saltwater therapy. It should be emphasized that despite a statistically significant decrease in the CRP level, its value before and after balneotherapy remained within the norm. Numerous data from the literature indicate that even low CRP values remaining within the normal range correlate with the risk of adverse cardiovascular events. In the first prospective epidemiological primary prevention study, the MRFIT study, which showed a strong association between high-sensitivity CRP (hs-CRP) levels and coronary artery disease (CAD) mortality in healthy middle-aged men with high cardiovascular risk, the mean CRP level among deceased subjects was 3.4 mg/L. However, in patients who suffered and survived, MI was at 2.7 mg/L and was not much different from the level of 2.9 mg/L in the control group [28]. Also in the MESA study, which analyzed 6722 people, hs-CRP was indicated as an independent biomarker differentiating people with and without future coronary events, and the cutoff limit was 3.76 mg/L, which was still within the normal range [29]. Most large-scale clinical trials have used an hs-CRP cutoff of 2 mg/L to determine increased cardiovascular risk [30]. This is crucial from the point of view of the risk of subclinical inflammation and adverse cardiovascular events because it turns out that even small increases in CRP result in increased cardiovascular risk. This is justified by the pathophysiology of CRP, which directly binds highly atherogenic, oxidized low-density lipoprotein cholesterol (LDL-chol), and both of these markers are present in lipid-containing plaques [31]. The role of CRP in atherosclerotic plaque formation is highly complex and is mediated by exerting a proatherogenic effect on many cells involved in atherosclerosis [32]. CRP may facilitate monocyte adhesion and migration into the blood vessel, which is a critical early stage of the atherosclerosis process [33]. In this way, it combines the concept of the inflammatory basis of atherosclerosis with lipid metabolism disorders, especially with increased levels of proatherogenic LDL-chol. In this context, balneotherapy in general [34], and in particular that which uses sulfur-containing water [9], may potentially reduce cardiovascular risk by lowering CRP levels. This thesis is additionally supported by quite numerous data showing that balneotherapy with salty sulfur–hydrogen sulfide waters reduces the level of LDL-chol [2,17,18]. Current research has confirmed a reduction in both of these cardiovascular risk factors, which suggests that cardiovascular risk will also be reduced. This is confirmed by the fact that no cardiovascular complications were noted during the observation period. 

Hang et al. showed that the cystathionine γ-lyase/H_2_S system increases the expression and the administration of NaHS facilitates the recruitment of neutrophils and increases the level of adhesion molecules: intercellular adhesive molucule-1, *p*-selectin, and S-selectin [35]. Increased H_2_S expression, therefore, plays a pro-inflammatory role in this aspect, especially in the acute stage and in neutrophil-related inflammation [36]. In contrast, growing evidence indicates that H_2_S does have anti-inflammatory effects. This thesis is supported by, for example, the observations of Mirandola et al., indicating that HS^−^ reduces the cellular cytotoxic response of peripheral blood lymphocytes, as well as their secretion of interleukin-2 (IL-2), thus deactivating the main local players of inflammatory responses. This study describes the pathophysiological rationale for S’s actions to the clinically known anti-inflammatory effects of S compounds [37]. Research conducted by Misztela on the use of sulfide and hydrogen sulfide baths in the treatment of patients with RA showed a significant reduction in CRP, as well as an increase in hemoglobin values, the level of which is usually lower in RA [38]. 

It was once quite arbitrarily established that CVD is a relative contraindication to balneotherapy with salty sulfide–hydrogen sulfide waters. The randomized study conducted by Oláh et al. provides important information regarding the safety of balneotherapy in diabetics with hypertension and obesity. It did not demonstrate any negative changes in antioxidant, inflammatory, or metabolic indicators [34]. However, it should be remembered that they only concern thermal water therapy. There are no data yet on the effect of sulfide–hydrogen sulfide salt water therapy on these parameters. Current research shows that in addition to reducing CRP, LDL-chol and DBP are significantly reduced. In this regard, the currently presented results, indicating a reduction in the analyzed key cardiovascular risk factors, are a valuable, innovative premise suggesting the broader qualification of patients for this form of therapy. 

### 4.4. Prothrombotic State

As noted earlier, after HSSB, FIBR levels escalated statistically significantly from 2.95 (±0.59) g/L to 3.23 (±1.23) g/L. Both values, however, remained within the normal range. Under physiological conditions, plasma concentrations of FIBR range from 2 to 4 g/L, and fibrinogen has a half-life of approximately 4 days. The increase in FIBR has a double meaning. First, elevated FIBR values are a known cardiovascular risk factor. FIBR is a risk factor for death or the recurrence of myocardial ischemia in patients with a previous coronary event and a predictor of accelerated coronary atherosclerosis [39,40]. Secondly, FIBR is an acute-phase protein whose concentration increases during inflammation and is a key regulator of inflammation in the disease [41]. Increasing evidence supports the important role of fibrin and FIBR and their degradation products in regulating the inflammatory response in numerous target tissues [42]. In most cases, the pro-inflammatory functions of fibrin(ogen) and its derivative peptides are associated with their ability to bind to and activate a wide range of immune cells through distinct ligand–receptor interactions. Importantly, these pro-inflammatory effects are a product of FIBR signaling through binding sites that are nonoverlapping with those involved in the coagulation cascade [41]. This results in an unfavorable cross-talk between coagulation and inflammation and its implications for human disease. Atherosclerosis is a chronic inflammatory condition that involves the gradual accumulation of lipids in the vessel wall; the infiltration of immune cells such as macrophages, T lymphocytes, and mast cells; and the local proliferation of vascular smooth muscle cells [43]. In advanced atherosclerotic plaques, the excessive accumulation of extracellular matrix proteins, such as fibrin and collagen, leads to the formation of a fibrous sheath and the local swelling of the vessel wall, which further limits the diameter of blood vessels and constitutes the nucleus of atherosclerotic lesions [43].

There are only a few publications on the changes in FIBR concentration after treatment with salty hydrogen sulfide waters. Most of these publications indicate that FIBR levels decrease after HSSB. Thus, contrary to the current results, Cencora et al. found that after an HSBB, the level of FIBR decreases [17]. Studies by Olas et al. showed that HS^−^, the source of which was NaHS, reduced the polymerization of purified FIBR and stimulated the lysis of fibrin in whole plasma [44]. Importantly, in vitro experiments indicate that both inactive and thrombin-activated platelets show a reduced ability to adhere to FIBR incubated with NaHS [45]. We should also remember the effect of H_2_S on blood rheology, which inhibits PLT activation and fibrin formation by dilating the vessel and reducing shear force [46]. The above-mentioned scientific premises, therefore, suggest that the use of substances that are a source of S^2−^ and HS^−^ ions may have a comprehensive antithrombotic effect, inhibiting both basic elements of this process—platelet activation and fibrin mobilization. Of course, this may potentially reduce the risk of thromboembolic complications.

One of the most important mechanisms underlying the pathophysiological reduction of cardiovascular events is the reduction in inflammation [3]. Therefore, the reduction in inflammatory markers observed in the current study, which is made manifest in a decrease in the CRP level, although statistically significant but still within the normal range, despite the increase in FIBR content, provides a net-beneficial anti-inflammatory effect of balneotherapy, improving the condition of the osteoarticular system. A slight increase while maintaining FIBR values in the normal range before and after HSSB should be treated as an expression of its hormetic effect. However, the potential prothrombotic effect of HSSB on the cardiovascular system seems to be at least neutral, and it cannot be ruled out that it is also indirectly beneficial by reducing the subclinical pro-inflammatory/prothrombotic state. Indirect evidence confirming these pathophysiological premises seems to be the unchanged level of biomarkers of both myocardial damage (cTnT) and HF (NT-proBNP), and, above all, the clinical observation of patients without adverse cardiovascular events during balneotherapy.

### 4.5. Cardiovascular Biomarkers

Due to the biologically active compounds S^2−^ and HS^−^ ion and colloidal S contained in it, salty sulfide-hydrogen sulfide waters penetrate the body, where biochemical, anti-inflammatory, anti-rheumatic, detoxifying, and anti-ischemic processes are activated. This form of treatment has always been accompanied by concerns about possible adverse effects on the cardiovascular system. This was the reason why many patients were not qualified for this therapy. The question often arose whether disqualifying HSSB patients was not too hasty. In this context, changes in cardiovascular biomarkers such as cTnT and NT-proBNP during HSSB are crucial to answering the objectives of the current study. Both parameters did not change statistically significantly after treatment with salty sulfide–hydrogen sulfide waters. The cTnT level before the balneotherapy session was 9.64 (±4.13) ng/L and it did not change statistically significantly after its completion, remaining at an almost identical level of 9.65 (±3.91) ng/L.

The issue of the impact of salty sulfide–hydrogen sulfide waters on the cardiovascular system is a problem that has been practically unexplored so far. There are no publications available regarding the impact of this form of therapy on potential myocardial damage. In this context, the assessment of cardiovascular safety is undoubtedly innovative from a scientific point of view. Certain premises confirming the current clinical results can be drawn from basic research indicating the beneficial effects of S^2−^ and HS^−^ ions on the heart. While high levels of H_2_S are extremely toxic, low levels are well tolerated and have potential cytoprotective and antioxidant effects [47]. Recent studies have confirmed that sulfur waters have a direct free radical scavenging effect, reduce ROS and reactive nitric species (RNS) released by human neutrophils during oxygen bursts, and protect against oxidative DNA damage, thus contributing to the therapeutic effects of these waters in the inflammation of the respiratory system [48,49,50]. H_2_S contained in hydrogen sulfide waters also blocks the production of inflammatory cytokines (IL-1β, TNF-α, IL-6, and IL-10) and counteracts the production of ROS and RNS by human monocytes [51]. Although there are unfortunately no data regarding the cardiovascular system, similar activity can be expected. This applies both to the beneficial antioxidant effect on the cardiovascular system and to the reaction at the cellular level. Activated monocytes are the basic morphotic pro-inflammatory element of unstable atherosclerotic plaque, contributing to complications in the form of ACS [52]. 

However, there are data indicating the cardioprotective effects of H_2_S from in vivo studies conducted on animals. Experimentally induced MI is one of the main models used to study the protective effects of H_2_S in ischemia [53]. The intravenous administration of Na_2_S 24 h before experimental cardiac ischemia reduced the size of myocardial infarction, serum cTn concentration, lipid hydroperoxide concentration, and the intensity of apoptosis [54]. The intraventricular injection of Na_2_S during reperfusion reduced the area of MI, cTn levels, inflammation, apoptosis, and the loss of left ventricular function. Na_2_S additionally enabled the maintenance of mitochondrial oxygen consumption at an unchanged level and the integrity of the myocardium after an episode of ischemia [55]. In a mouse model, the administration of water with HS^−^ ions (NaHS) for 4 weeks after MI allowed for the inhibition of the deepening of cardiac dysfunction and resulted in an increase in the number of collaterals of the coronary circulation [56]. Genetically modified mice lacking the H_2_S-producing enzyme CSE (CSE−/−) exhibit elevated oxidative stress, dysfunctional endothelial NOS, diminished NO levels, and exacerbated myocardial and hepatic ischemia/reperfusion injury [8]. The reduced production of endogenous H_2_S that is seen in mice lacking the CSE gene (CSE−/−) is also associated with accelerated atherosclerosis [57]. The latter publication also showed that the rate of atherosclerosis in these animals could be reduced by substituting SH- with NaHS [57]. Researchers suggest that H_2_S-mediated cytoprotective signaling during ischemia/reperfusion injury is largely dependent on the activation of endogenous NOS and, through this, an increase in NO production [8]. However, the mechanisms of H_2_S-induced protection remain unclear, although many different pathophysiological pathways have been investigated. H_2_S appears to cause numerous and diverse physiological and pathophysiological effects. Currently, it is not possible to formulate a universal hypothesis that explains all the effects of these models [53]. However, there is a consensus that the lack of H_2_S contributes to the accelerated progression of atherosclerosis [58].

The analysis of the relationship between the cTnT level before balneological treatment revealed that it remains in a statistically significant correlation with the level of LDL-chol, CREAT, GFR, and NT-proBNP. A similar analysis carried out after a sanatorium treatment with salty sulfide–hydrogen sulfide waters showed that TCHOL, LDL-chol, CREAT, GFR, and NT-proBNP were significantly correlated with the level of cTnT. The significant correlation of cTnT with LDL-chol observed both before and after balneotherapy is a potential association of a biomarker of myocardial damage and atherogenic and pro-inflammatory molecules such as LDL-chol. The key importance of inflammation for the initiation and progression of atherosclerosis, and consequently for cardiovascular events that are usually a consequence of complications of atherosclerotic plaque destabilization, is well known and established [31]. Highly atherogenic, oxidized LDL-chol is directly bound by CRP, stimulating the pro-inflammatory state that is present in lipid-laden plaques [31]. Although at the stage of univariate correlations the relationship between cTnT and LDL-chol seems convincing, it is not confirmed in the multiple regression analysis, where LDL-chol is no longer a marker independently associated with cTnT both before and after balneotherapy. Additionally, it should be remembered that while the cTnT level does not change statistically significantly over the course of observation, the LDL-chol level decreases significantly. The negative nature of the correlation of cTnT with LDL-chol in the context of extensive data from the literature indicating a positive association of cTnT with LDL-chol [59] and the lack of correlation in the multiple regression analysis ultimately calls into question the value of this correlation and suggests considering it as a statistical error. Finally, the positive correlation of cTnT with NT-proBNP is consistent with the commonly observed fact of the mutual positive correlation of these biomarkers. This applies even to a healthy population, where the very detection of cTnT, although in most cases still at levels normal for a healthy population, was associated with an increased level of NT-proBNP [60]. Furthermore, apparently, healthy adults with detectable levels of cTnT or minimally elevated NT-proBNP levels in long-term follow-up have been found to be at increased risk of death. However, people with minimally elevated concentrations of both cTnT and NT-proBNP are at even greater risk, and the increased risk persists for years [60].

In summary, the results of the current studies indicate that HSSB has at least a neutral effect on potential myocardial damage. Moreover, numerous and consistent data in the literature provide evidence from basic science, additionally confirmed through in vivo animal studies, consistently pointing to the beneficial cardioprotective effects of various biologically active forms of S. Therefore, it seems that treatment with salty sulfide–hydrogen sulfide waters is at least safe for the circulatory system. Moreover, numerous pathophysiological premises create reasonable hopes for their potential beneficial cardioprotective effect. Following this line of thought, we should be critical of excessive restrictions in qualifying patients with CVDs for HSSB, and we should certainly exercise great restraint in hastily disqualifying patients from this form of therapy. 

Saltwater sulfide–hydrogen sulfide therapy also had no effect on the level of HF biomarkers, i.e., NT-proBNP. It remained unchanged at a statistically significant level before and after balneotherapy: 137.41 (±176.52) vs. 142.89 (±182.82) pg/mL. It is worth noting that the levels were, although only slightly, above the values considered in the standards as borderline for the diagnosis of HF (125 pg/mL) [21]. 

Studies on the use of sulfur and hydrogen sulfide waters in HF are few and, according to the available literature, only concern animal studies. Studies conducted on an HF model in rats caused by diabetic cardiomyopathy in the course of streptozotocin-induced diabetes showed an increased expression of the following genes: transforming growth factor-β1 (TGF-β1), procollagen-1, and matrix metalloprotenase-2 MMP-2 [61]. The increased expression of profibrogenic genes and an increase in collagen, as reflected by an increase in hydroxyproline levels, ultimately lead to cardiac fibrosis and demonstrate their obvious implication in the pathogenesis of diabetic cardiomyopathy. Mineral water with S salts, including NaHS as an HS^−^ donor, used in the examined animals, probably counteracted the increased expression of profibrogenic apoptotic substances by increasing the level of cardiac glutathione [61]. The observed beneficial antifibrotic and antiapoptotic effects of S mineral water and NaHS in the hearts of diabetic rats suggest potential therapeutic options for diabetic cardiomyopathy. The results of the cited study suggest that H_2_S donors may be considered as a novel and applicable approach in preventing or treating cardiac fibrosis associated with diabetes [61].

In a model of HF caused by arterial hypertension, it was clearly demonstrated that H_2_S slows down the progression to unfavorable left ventricular remodeling, i.e., cardiac remodeling, and induces angiogenesis in the myocardium [62]. The importance of H_2_S in the pathogenesis of HF is suggested by studies in which the limited cardiac overexpression of CSE in mice resulted in increased endogenous H_2_S production and protection against ischemia-induced HF with an accompanying reduction in mortality [62]. In contrast, CSE knockout resulting in reduced reactive sulfur production in mouse models of HF showed worsening myocardial function and larger MI size [63]. Other data on HF from animal studies using active sulfur donors indicate its beneficial anti-remodeling effect in a model of MI in mice drinking water containing NaSH [56]. Research by Qipshidze et al. revealed that left ventricle ejection fraction (LVEF) following experimental MI decreased from 64 (±0.4)% to 28 (±0.4)% [56]. However, if exogenous NaHS as an H_2_S/HS^−^ donor was administered at the time of MI and for the next 4 weeks, the extent of MI was significantly reduced, as LVEF in these mice was reduced much less—only to an average level of 47 (±0.3)% [56]. This is undoubted evidence of limiting unfavorable post-infarction remodeling and even of retrograde left ventricular remodeling. A histopathological examination further showed that H_2_S treatment alleviated the formation of left ventricle fibrosis in the treated group compared to the NaHS-untreated control group. [56]. Other studies have shown that the key importance of H_2_S in the prevention of HF is its protective effect against ischemia-induced fibrosis and ischemia-induced inflammation [64]. The induction of hypometabolism by H_2_S is also important, which provides additional short-term protection against myocardial necrosis [64]. Mishra et al. found that cardiac fibrosis and apoptosis in chronic HF were reversed via the administration of H_2_S, which was associated with a reduction in oxidative and proteolytic stress [65]. Research by Huang et al. indicates that the antifibrotic effect of H_2_S, manifested in a reduction in the amount of cardiac connective tissue and collagen content, occurs through the inhibition of intracardiac Ang-II activity [66]. 

Most studies on H_2_S have used inorganic sulfide salts such as sodium hydrogen sulfide (NaHS) and sodium sulfide (Na_2_S). These compounds increase the concentration of H_2_S/HS^−^ due to the dissociation and pH-dependent balance between HS- and H_2_S in aqueous solutions. A similar thing happens in the case of treatment with salty sulfide–hydrogen sulfide waters, where individual forms of sulfur remain in a dynamic balance. NaHS has been shown to have a protective effect in a number of experimental models of ischemia-reperfusion injury. Several preclinical and clinical studies have also been carried out using various reactive sulfur donors. Thus, sodium polysulfonate, a new long-acting prodrug that is a source of H_2_S (substance SG1002), in preclinical studies reduces remodeling and dysfunction of the left ventricle in an HF model caused by pressure overload [62]. The administration of SG1002 significantly increased both H_2_S and NO levels and reduced numerous disease markers [62]. A clinical trial with Sulfagenix (commercial name of the substance SG1002) revealed for the first time its safety in both healthy people and patients with HF. Moreover, this new H_2_S-releasing prodrug has been shown to increase the amount of bioavailable H_2_S and its free metabolites such as S in humans [67]. Sulfane S is a reactive form of S and has regulatory functions in various biological systems. Most of the beneficial biological effects of H_2_S are attributed to this form of S [68]. SG1002 also significantly increases NO bioavailability, as measured in plasma nitrite levels. The results of this study are promising and indicate that the use of SG1002 in the treatment of patients with HF is worth further investigation. A larger, placebo-controlled phase II study is currently being designed in patients with HF to investigate SG1002’s ability to raise H_2_S levels, reduce oxidative stress, and ultimately improve cardiac function. 

The analysis of the relationship between the concentration of NT-proBNP before treatment with salty sulfide-hydrogen sulfide water revealed that it correlates statistically significantly with CRP and cTnT. The multiple regression analysis showed that before balneotherapy, none of the laboratory parameters were independently associated with the level of NT-proBNP. The correlation analysis performed after balneological treatment showed that TGC and cTnT are significantly related to the level of NT-proBNP. The multiple regression analysis revealed that none of the parameters were independently associated with NT-proBNP levels after HSBB. When commenting on these related issues, it is worth paying attention to two threads. The association of NT-proBNP with CRP before treatment raises an obvious association with the potential association of subclinical inflammation with HF. This, of course, raises concerns about whether the hormetic effect of HSSB stimulates a pro-inflammatory state and, thus, indirectly increases the potential threat of HF stimulation. However, it is known that subclinical pro-inflammatory states and even more overt clinical inflammations are known factors stimulating the progression of HF [69,70]. However, based on all the results obtained, it seems that these fears are scientifically and even more clinically unjustified. Firstly, the level of NT-proBNP did not change after HSSB and at the same time remained at a level only slightly above the norm during the entire observation. Secondly, the objective inflammation indicator, CRP, which has been proven by numerous studies, remained at the normal level before and after the therapy, but after its completion, it was statistically significantly reduced. Thus, global pro-inflammatory readiness (CRP) decreased and HF did not progress.

The second topic requiring comment is the relationship between NT-proBNP and cTnT. First of all, it should be noted that the values of both these cardiac markers remained within the normal range (cTnT) or close to the normal range (NT-proBNP) throughout the study. Secondly, as noted in the earlier discussion of the relationship between cTnT and NT-proBNP, even with normal or slightly elevated values in a population considered healthy, these biomarkers remain positively correlated with each other [60]. It should also be remembered that the source of cTn release is not only tissue necrosis, which usually causes large increases. Another rarely realized reason is a slight “seepage” of cTn as a result of apoptosis, i.e., natural cell death. [71,72,73,74]. One of the reasons for apoptosis may be increased stress on the walls of the heart chambers, especially the left ventricle, related to the pressure overload characteristic of HF. A sensitive marker of stress, and not necessarily pathological, is NT-proBNP, the level of which, elevated above the established norms, is, of course, only one of the elements of the diagnosis of HF [21]. 

To summarize, the lack of changes in the levels of the HF biomarker NT-proBNP assessed in the current study indicates the high safety of HSSB. Although there are limited data from the literature, conducted exclusively on in vivo animal models, the pathophysiological premises arising from them, and the current results indicate that this form of balneotherapy has not only neutral but even beneficial effects also in the area of HF. This thesis is supported by the already available results of clinical trials with bioactive sulfur donors, indicating its beneficial effect on HF [68]. Therefore, the neutral effect of saltwater sulfide–hydrogen sulfide therapy on markers of myocardial damage (cTnT) together with the lack of changes in the concentration of the HF biomarker (NT-proBNP) allows us to conclude that this therapy is safe in the context of adverse cardiovascular events. However, to verify these initial favorable study results in the OAD patient population, controlled studies are necessary in selected populations, i.e., those with ischemic heart disease and HF. 

### 4.6. Limitations of the Study

Although the study group was very uniform in terms of indications for sanatorium rehabilitation because according to them it was dominated by patients with SOA and OA, just over 50% (51 patients) of the study population had CVDs at the same time. This evidently does not allow for the assessment of the impact of balneotherapy using salty sulfide–hydrogen sulfide water on the cardiovascular system in patients with CVDs. However, it is worth recalling and emphasizing that the aim of the study was to assess the impact of this form of balneotherapy on selected indicators of inflammation and markers of the cardiovascular system in the general population of patients with OADs, not CVDs. The fact that the study group included, by chance, over 50% with some CVD, gives this assessment an additional scientific and practical value in relation to patients with CVDs. However, it does not allow for full conclusions about the impact of this form of therapy on the cardiovascular system in patients with CVDs, but only, as noted in the title, on markers of the cardiovascular system in the population treated with salty sulfide–hydrogen sulfide waters. However, so far, there are no studies in the literature on the effect of HSSB on cardiovascular markers. Even more so, there is no such research in the group of patients with CVDs, which currently still constitutes a limitation in qualifying for HSSB. It should be remembered that qualifications for the current study had to be based on current standards, taking into account applicable contraindications from the cardiovascular system. Secondly, it was not a randomized clinical trial dedicated to the impact of HSSB on the cardiovascular system in patients with CVDs, because that would evidently require other clinical and legal conditions and the establishment of endpoints commonly analyzed in clinical trials. It is important, however, that in the analyzed group, where comorbid CVDs accounted for over 50%, no cardiovascular complications were recorded. Moreover, markers of myocardial damage (cTnT) and HF did not change significantly over the course of follow-up and always remained at normal (cTnT) or minimally elevated levels (NT-proBNP). Together, this provides an additional incentive to plan dedicated studies for CVD patients undergoing HSSB to verify the promising results of current studies.

## 5. Conclusions

HSBB resulted in statistically significant improvement in a subclinical pro-inflammatory state.

HSBB has a beneficial effect in modifying key cardiovascular risk factors by reducing LDL-C levels and DBP values. 

HSBB has a neutral effect on cardiovascular ischemia/injury.

Despite slightly elevated baseline levels of the biochemical marker of HF (NT-proBNP), HSBB causes no further increase in this marker.

The use of HSBB in patients with OAD has either a neutral effect or a potentially beneficial effect on the cardiovascular system, which may constitute grounds for further studies to verify the current cardiovascular contraindications for this form of therapy.

## Figures and Tables

**Table 1:** Basic demographic data in the entire study group.

Parameter	N	Mean Value	Minimum	Maximum	Deviation Std.
age [years].	97	67.32	43.00	85.00	7.59
body weight [kg]	97	74.97	57.00	115.00	11.98
BMI [kg/m^2^]	97	27.34	21.60	41.10	3.74
height [cm]	97	165.43	145.00	188.00	8.06
BSA [m^2^]	97	1.82	1.56	2.26	0.16

Abbreviations: BMI—body mass index; BSA—body surface area.

**Table 2:** The impact of balneological treatment on the following parameters: SBP, DBP, HR, and biochemical markers in the study group.

Parameter	Before Balneotherapy			After Balneotherapy		
	n	Mean Value	Deviation Std.	Mean Value	Deviation Std.	*p*
SBP [mmHg]	97	139.90	19.69	136.11	19.99	0.068
DBP [mmHg]	97	77.79	8.69	74.16	9.02	<0.00001
HR [b/min]	97	74.47	11.76	72.93	10.74	0.103
FIBR [g/L]	97	2.95	0.59	3.23	1.23	0.021
TCHOL [mg/dL]	97	213.43	44.23	211.89	41.38	0.547
TGC [mg/dL]	97	121.37	56.30	142.92	69.43	0.00002
HDL-chol [mg/dL]	97	59.58	15.66	57.21	14.28	0.00019
LDL-chol [mg/dL]	97	129.36	40.67	123.74	36.14	0.016
CREAT [mg/dL]	97	0.74	0.14	0.73	0.13	0.447
GFR [ml/min/1.73 m^2^]	97	83.21	18.53	84.11	17.92	0.414
UA [mg/dL]	97	5.66	2.96	5.38	1.24	0.306
CRP [mg/L]	97	2.70	3.60	2.06	1.91	0.025
NT-proBNP [pg/mL]	97	137.41	176.52	142.89	182.82	0.477
cTnT [ng/L]	97	9.64	4.13	9.65	3.91	0.949

Abbreviations: SBP—systolic blood pressure, DBP—diastolic blood pressure, HR—heart rate, FIBR—fibrinogen, TCHOL—total cholesterol, TGC—triglycerides, HDL-chol—high-density lipoprotein cholesterol, LDL-chol—low-density lipoprotein cholesterol, CREAT—creatinine, GFR—glomerular filtration rate, UA—uric acid, CRP—C-reactive protein, cTnT—cardiac troponin T, and NT-proBNP—N-terminal pro-B-type natriuretic peptide.

**Table 3:** The relationship between the cTnT level and other laboratory parameters before and after balneotherapy in the study group.

Parameter	cTnT Concentration [ng/L]					
	Before Balneotherapy			After Balneotherapy		
	R	*p*	n	R	*p*	n
FIBR [g/L]	0.185	0.080	97	−0.118	0.271	97
TCHOL [mg/dL]	−0.199	0.059	97	−0.231	0.029	97
TGC [mg/dL]	−0.065	0.538	97	−0.169	0.115	97
HDL-chol [mg/dL]	0.129	0.225	97	0.030	0.779	97
LDL-chol [mg/dL]	−0.239	0.023	97	−0.234	0.027	97
CREAT [mg/dL]	0.295	0.003	97	0.357	0.0003	97
GFR [mL/min/1.73 m^2^]	−0.313	0.009	97	−0.349	0.0019	97
UA [mg/dL]	0.038	0.718	97	0.207	0.051	97
CRP [mg/L]	−0.006	0.948	97	0.119	0.265	97
NT-proBNP [pg/mL]	0.247	0.013	97	0.219	0.040	97

Abbreviations: cTnT—cardiac troponin T, FIBR—fibrinogen, TCHOL—total cholesterol, TGC—triglycerides, HDL-chol—high-density lipoprotein cholesterol, LDL-chol—low-density lipoprotein cholesterol, CREAT—creatinine, GFR—glomerular filtration rate, UA—uric acid, CRP—C-reactive protein, and NT-proBNP—N-terminal pro-B-type natriuretic peptide.

**Table 4:** Parameters independently associated with cTnT before balneotherapy in the study group. Multiple regression analysis; model: R = 0.445; R^2^ = 0.198; *p* < 0.00745.

Dependent Variable	Independent Variable	β	B	Deviation Std.	*p*
cTnT [ng/L]	LDL-chol [mg/dL]	−0.112	−0.011	0.010	0.272
	CREAT [mg/dL]	0.226	6.551	3.007	0.032
	GFR [ml/min/1.73 m^2^]	−0.252	−3.533	4.384	0.012
	UA [mg/dL]	−0.047	−0.066	0.142	0.643
	CRP [mg/L]	−0.115	−0.129	0.114	0.261
	NT-proBNP [pg/mL]	0.171	0.003	0.002	0.108

Abbreviations: cTnT—cardiac troponin T, LDL-chol—low-density lipoprotein cholesterol, CREAT—creatinine, GFR—glomerular filtration rate, UA—uric acid, CRP—C-reactive protein, and NT-proBNP—N-terminal pro-B-type natriuretic peptide.

**Table 5:** Parameters independently associated with cTnT after balneotherapy in the study group. Multiple regression analysis; model: R = 0.411; R^2^ = 0.169; *p* < 0.00505.

Dependent Variable	Independent Variable	β	B	Deviation Std.	*p*
cTnT [ng/L]	TCHOL [mg/dL]	−0.032	−0.003	0.017	0.862
	LDL-chol [mg/dL]	−0.101	−0.010	0.019	0.583
	CREAT [mg/dL]	0.295	8.469	2.930	0.004
	GFR [ml/min/1.73 m^2^]	0.274	5.643	2.943	0.001
	NT-proBNP [pg/mL]	0.092	0.001	0.002	0.364

Abbreviations: cTnT—cardiac troponin T, TCHOL—total cholesterol, LDL-chol—low-density lipoprotein cholesterol, CREAT—creatinine, GFR—glomerular filtration rate, and NT-proBNP—N-terminal pro-B-type natriuretic peptide.

**Table 6:** The relationship between the NT-proBNP level and other laboratory parameters before and after balneotherapy in the study group.

Parameter	NT-proBNP Concentration [pg/mL]					
	Before Balneotherapy			After Balneotherapy		
	R	*p*	n	R	*p*	n
FIBR [g/L]	0.052	0.623	97	−0.026	0.808	97
TCHOL [mg/dL]	−0.106	0.316	97	−0.119	0.266	97
TGC [mg/dL]	−0.158	0.135	97	−0.217	0.042	97
HDL-chol [mg/dL]	0.106	0.315	97	0.092	0.393	97
LDL-chol [mg/dL]	−0.112	0.290	97	−0.068	0.527	97
CREAT [mg/dL]	0.155	0.143	97	0.115	0.282	97
GFR [ml/min/1.73m^2^]	−0193	0.175	97	−0.163	0.343	97
UA [mg/dL]	−0.033	0.753	97	−0.042	0.693	97
CRP [mg/L]	0.248	0.013	97	0.006	0.953	97
cTnT [ng/L]	0.271	0.0095	97	0.218	0.041	97

Abbreviations: NT-proBNP—N-terminal pro-B-type natriuretic peptide, FIBR—fibrinogen, TCHOL—total cholesterol, TGC—triglycerides, HDL-chol—high-density lipoprotein cholesterol, LDL-chol—low-density lipoprotein cholesterol, CREAT—creatinine, GFR—glomerular filtration rate, UA—uric acid, CRP—C-reactive protein, and cTnT—cardiac troponin T.

**Table 7:** Parameters independently associated with NT-proBNP before balneotherapy in the study group. Multiple regression analysis; model: R = 0.428; R^2^ = 0.184; *p* < 0.00673.

Dependent Variable	Independent Variable	β	B	Deviation Std.	*p*
NT-proBNP [pg/mL]	TGC [mg/dL]	−0.099	−0.320	0.321	0.322
	cTnT [ng/L]	0.147	6.318	4.417	0.156

Abbreviations: NT-proBNP—N-terminal pro-B-type natriuretic peptide, TGC—triglycerides, and cTnT—cardiac troponin T.

**Table 8:** Parameters independently associated with NT-proBNP after balneotherapy in the entire study. Multiple regression analysis; model: R = 0.495; R^2^ = 0.245; *p* < 0.00061.

Dependent Variable	Independent Variable	β	B	Deviation Std.	*p*
NT-proBNP [pg/mL]	TGC [mg/dL]	−0.090	−0.247	0.292	0.401
	cTnT [ng/L]	0.102	4.756	4.612	0.305

Abbreviations: NT-proBNP—N-terminal pro-B-type natriuretic peptide, TGC—triglycerides, and cTnT—cardiac troponin T.

## Data Availability

The data used to support the findings of this study are available from the corresponding author upon request.
